# Research on Inlet and Outlet Structure Optimization to Improve the Performance of Piezoelectric Pump

**DOI:** 10.3390/mi11080735

**Published:** 2020-07-29

**Authors:** Xiaolong Zhao, Dingxuan Zhao, Jiantao Wang, Tao Li

**Affiliations:** 1 School of Mechanical Engineering, Yanshan University, Qinhuangdao 066004, China; zxlysu@139.com (X.Z.); zdx-yw@ysu.edu.cn (D.Z.); 2 School of Vehicle and Energy, Yanshan University, Qinhuangdao 066004, China; 3 Postdoctoral Mobile Station of Mechanical Engineering, Yanshan University, Qinhuangdao 066004, China; 4 School of Mechanical Science and Engineering, Jilin University, Changchun 130025, China; lwzysci@yeah.net

**Keywords:** piezoelectric pump, structure optimization, dynamic load, elastic inlet and outlet

## Abstract

As piezoelectric pumps are used in more fields, they are gradually failing to meet the application requirements due to their low output performance. Therefore, improving the output performance of piezoelectric pumps helps to expand their applications. This paper argued that the dynamic load of liquid in the inlet and outlet pipelines was an important factor that weakened the performance of piezoelectric pumps. Therefore, in order to reduce the dynamic load, it was proposed to replace the conventional piezoelectric pump inlet and outlet by an elastic inlet and outlet. After introducing the structure and working principle of elastic inlet and outlet, the mechanism of reducing the dynamic load by elastic inlet and outlet was analyzed. Then, the influence of the elastic cavity height on the performance of the piezoelectric pump was studied from both fluid simulation and theoretical analysis. Finally, several prototypes were made. The effectiveness of the elastic inlet and outlet on improving the performance of the prototype and the effect of the elastic cavity height on the performance of the prototype were tested, respectively. The test results showed that the elastic inlet and outlet effectively improved the flow rate and output backpressure without increasing the maximum output backpressure. The maximum flow rate of the pump system without load was increased by 36%. In addition, the elastic cavity height adversely affected the flow rate and output backpressure of the prototypes, but had no effect on the maximum output backpressure. In summary, the elastic inlet and outlet can effectively increase the output performance of the piezoelectric pump, but the design height should be appropriately reduced.

## 1. Introduction

Piezoelectric pumps are microfluidic machinery. Piezoelectric pumps have the advantages of simple structure, high driving strength, easy miniaturization, no magnetic influence and low noise [1,2,3,4,5], etc. They can be applied to biomedical [6,7,8], microelectromechanical systems [9,10] and fuel cells [11,12]. However, the current output performance of piezoelectric pumps is generally low, which limits their application in many fields. Therefore, it is of great significance to improve the performance of piezoelectric pumps for expanding their application fields.

In order to improve the output performance of piezoelectric pumps, a lot of research has been undertaken by scholars. These studies can be grouped into the following five main research areas. The first research area is how to improve the output performance of piezoelectric pump drive units. Some researchers have improved the output of piezoelectric pumps by optimizing the structure of the drive unit to improve the output displacement or output force of the drive unit. In 2015, Chen et al. [13] designed a U-shaped resonator to improve the performance of the piezoelectric pump. The U-shaped structure improved the stress distribution and output displacement of the actuator. In 2016, Wang et al. [14] proposed a rectangular piezoelectric vibrator that can effectively release the vibrating constraints of the vibrator, and enlarge its center output displacement. In 2019, Wang et al. [15] proposed a square piezoelectric vibrator with a flexible support that was used as the driving unit of the pump. The vibrator with a flexible support increased the volume change of the pump chamber, thus improving the performance of the piezoelectric pump. The second research area is the optimization of check valves. Check valves have a hysterical response during high-frequency operation, which results in a degradation of the output performance of piezoelectric pumps. In order to reduce the impact of the hysteresis response of the check valve on the performance of piezoelectric pumps, researchers have designed some high-frequency response check valves. In 2005, Li et al. [16] proposed a robust passive high frequency high pressure micro check valve with novel cross-patterned microvalve flap. The frequency of this valve reached 10 kHz. In 2006, Yang et al. [17] designed planar micro-check valves exploiting large polymer compliance. Testing results show that the check valves can achieve a diodicity up to 10^5^. In 2018, Yang et al. [18] proposed a technique of check valve improvement for high-frequency and high flow rate piezoelectric pumps. The high-frequency performance of the valve was improved by adding in a blocking edge over polydimethylsiloxane (PDMS) film check valve. At present, the existing research on high-frequency response check valves has become mature. The third research area is the optimized design of valveless piezoelectric pumps. The researchers designed some valveless piezoelectric pumps to eliminate the detrimental effect of check valves on piezoelectric pumps, thus improving the performance of the piezoelectric pump. In 2018, Munas et al. [19] designed a valveless piezoelectric pump with a cross junction. The cross junction is generated by a nozzle jet attached to a pump chamber and the intersection of two inlet channels and an outlet channel, respectively. The structure of cross junction facilitated a complete fluidic path throughout the system. In 2020, Van et al. [20] proposed a synthetic jet valveless pump in which the pump chamber is sealed on one side and connected to an emitting nozzle at another side. The valveless pump used a lead zirconate titanate (PZT) diaphragm to actuate a synthetic jet. In 2019, Matteo et al. [21] proposed a predictive approach for synthetic jet formation, which can help the designer to manufacture a synthetic jet pump having the desired performance. The fourth research area is the structural optimization of pump chamber and check valve. Scholars have optimized the structure of the pump chamber and check valve to reduce power loss and thus improve output performance. In 2016, Zhang et al. [22] proposed a single active-chamber piezoelectric pump with multiple passive check valves to prevent the backward flow of piezoelectric pumps. The pump could provide accurate flow rate and improve the anti fatigue and wear of the check valve. In 2019, Farshchi et al. [23] investigated the fluid dynamic response and the fluid–structure interaction during different stages of the working cycle by means of a 3-D finite element (FE) model. Their research work provides a reference for the structural optimization of piezoelectric pumps. To improve the stability and reliability of the piezoelectric pump, in 2015, Chen et al. [24] analyzed the cause of the air block phenomenon from the structure of a wheel check valve and the optimal combination of the wheel check valve structure is obtained within the samples: as the thickness is 0.02 mm, the diameter ratio is 1.2, the wheel check valve opening height gets 252 μm, and within the given bubble volume, the air block probability is less than 2%. The fifth research area is the joint application of several piezoelectric pumps. The researchers connected several piezoelectric pumps in series or parallel and then controlled them sequentially, greatly improving the output performance of the piezoelectric pump units [25,26,27,28,29,30]. It needs to be added that, in addition to these five main research directions, there are also many other studies which have contributed to the performance improvement of piezoelectric pumps.

The aforementioned studies have made significant contributions to improving the output performance of piezoelectric pumps. However, unlike the above studies, this paper attempts to improve the output performance of piezoelectric pumps by reducing the dynamic loading of the liquid in the inlet and outlet pipelines. In this paper, it is proposed that the dynamic load of the liquid in the inlet and outlet pipelines is one of the most important reasons for reducing the output performance of piezoelectric pumps. When the piezoelectric pump is operating, the liquid in the inlet and outlet pipelines is in a state of high-frequency vibration. This vibration is manifested in the pulsation of flow velocity. The dynamic load of the liquid is the inertial force generated by the high-frequency vibration of the liquid. Obviously, the dynamic load increases the power loss of the pump system, which is an important factor affecting the output performance of the piezoelectric pump. In order to reduce the dynamic load, the elastic cavity is added at the connection between the inlet pipeline and the pump chamber as well as the connection between the outlet pipeline and the pump chamber. As a result, the traditional rigid inlet and outlet are modified into the flexible inlet and outlet. The elastic cavity can cut off the rigid connection between the liquid in the pump chamber and the liquid in the pipelines. At this point, the elastic cavity is similar to the elastic damper in a mechanical vibration system, which can effectively reduce vibration. So the amplitude of the liquid in the pipelines is reduced. The elastic cavity smooths the flow rate of the liquid in the pipelines and reduces the dynamic load of the liquid. The advantage of the scheme proposed in this paper is that it has a simple structure and can effectively improve the output performance of the piezoelectric pump without increasing the input power and volume of the piezoelectric pump.

In this paper, firstly, the structure and working principle of the elastic inlet and outlet were introduced. Secondly, a theoretical analysis of the mechanism of the elastic inlet and outlet reducing the dynamic load was carried out. Then, the effect of the elastic cavity height on the output performance of the piezoelectric pump was studied from both fluid simulation and theoretical analysis. Finally, several prototypes were made and tested.

## 2. Structure and Working Principle of the Elastic Inlet and Outlet

**Figure 1:**
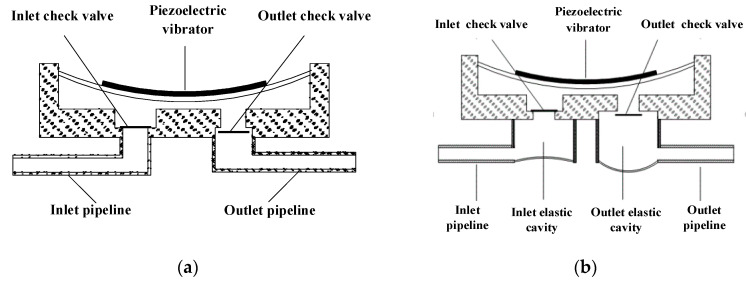
Schematic diagram of inlet and outlet structure optimization. (**a**) Piezoelectric pump with traditional inlet and outlet, (**b**) piezoelectric pump with elastic inlet and outlet.

First, the working principle of the piezoelectric pump with traditional inlet and outlet is introduced. The structural diagram of the piezoelectric pump with traditional inlet and outlet is shown in Figure 1a. When the piezoelectric vibrator is driven upward by the voltage, the volume of pump chamber increases and the chamber pressure decreases. At the same time, the outlet check valve is closed and the inlet check valve is open. So the liquid in the inlet pipeline is drawn into the pump chamber. When the piezoelectric vibrator is driven downward by the voltage, the volume of pump chamber decreases and the chamber pressure increases. At the same time, the outlet check valve is open and the inlet check valve is closed. So the liquid in the pump chamber is squeezed into the outlet pipeline. Due to the high-frequency up and down motion of piezoelectric vibrator, the liquid in the inlet and outlet pipelines vibrates synchronously with the liquid in the pump chamber.

**Figure 2:**
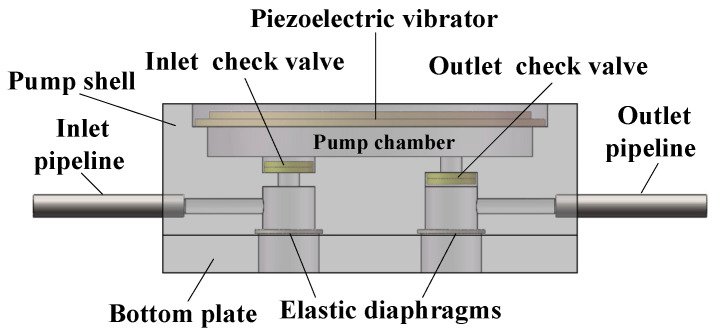
The structure of piezoelectric pump with elastic inlet and outlet.

In this paper, the structural optimization of the inlet and outlet is to add an elastic cavity outside the inlet and outlet check valves, respectively. As a result, the rigid structure of the inlet and outlet is transformed into an elastic structure. This inlet and outlet are referred to as elastic inlet and outlet in the paper. The structural diagram of the piezoelectric pump with elastic inlet and outlet is shown in Figure 1b. The bottom structure of the elastic cavity should be an elastic diaphragm with high elastic deformation capacity. In this paper, the elastic diaphragm material is silicone. The structure of the elastic inlet and outlet is shown in Figure 2.

**Figure 3:**
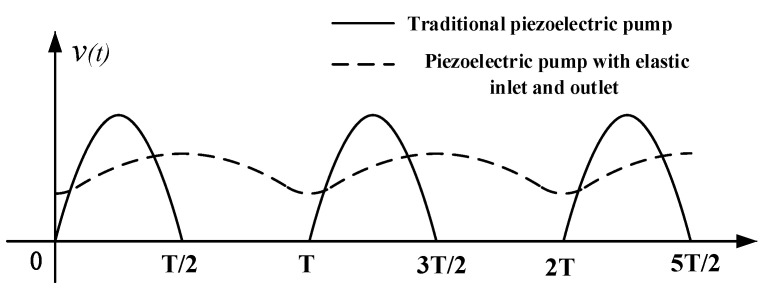
Velocity curves of liquid in the pipelines of the traditional piezoelectric pump and the piezoelectric pump with elastic inlet and outlet.

When a piezoelectric pump is working, the liquid in the pump chamber and pipelines is driven by the piezoelectric vibrator to produce high-frequency vibration. According to the working characteristics of the piezoelectric pump, the vibration of the liquid in pipelines and pump chamber can be approximated as sinusoidal vibration. Due to the role of the check valve, the liquid in the pipelines only vibrates in one direction. The elastic cavity is similar to the vibration buffer in the mechanical vibration system. The elastic cavity cuts off the rigid connection between the liquid in the pump chamber and the liquid in the pipelines. As a result, the velocity fluctuations of the liquid in the pipelines are suppressed. Vibration speed comparison curves of liquid in the pipelines of the traditional piezoelectric pump and the piezoelectric pump with elastic inlet and outlet are shown in Figure 3. The elastic cavity smooths the flow velocity of the liquid in the pipelines and reduces the dynamic load of the liquid in the pipelines.

## 3. Mechanism Analysis of Elastic Inlet and Outlet to Reduce Liquid Dynamic Load

The dynamic load of liquid in the inlet pipeline follows the identical generation mechanism to that in the outlet pipeline, so only the generation mechanism of the dynamic load of liquid in the outlet pipeline is explained. In order to simplify the analysis process, we made some assumptions. Assume that the liquid is incompressible and ignore the liquid flow resistance at the check valves. So we regard the liquid in pump chamber and pipeline as rigid connection. The liquid in the pump chamber was considered as the equivalent lumped mass. When the piezoelectric actuator is driven by a sinusoidal signal with the frequency *f*, the vibration displacement of the liquid in the pump chamber is defined as:

(1) $$X_{1}=\Phi_{1}\sin(2\pi ft)$$

In Equation (1), Φ_1_ is the maximum amplitude of the liquid in the pump chamber. Liquid in the pump system is considered incompressible. When the piezoelectric pump without elastic inlet and outlet discharges liquid, the liquid in the pump chamber and the outlet pipeline will be rigidly connected. Thus, the vibration displacement of the liquid in the outlet pipeline is expressed as:

(2) $$X_{2}=\Phi_{2}\sin(2\pi ft)$$

In Equation (2), Φ_2_ denotes the maximum amplitude of the liquid in the outlet pipeline. The flow of liquid from the pump chamber to the outlet pipeline fits the mass conservation theory, so *X*_1_ and *X*_2_ satisfy the following equation:

(3) $$A_{1}X_{1}=A_{2}X_{2}$$

In Equation (3), *A*_1_ denotes the cross-sectional area of the pump chamber, and *A*_2_ is the cross-sectional area of the outlet pipeline. Thus, the dynamic load of the liquid in the outlet pipeline is written as:

(4) $$F_{2}=m_{2}{\overset{••}{X}}_{2}=m_{2}\frac{A_{1}}{A_{2}}\Phi_{1}{\left(2\pi f\right)}^{2}\sin(2\pi f)$$

In Equation (4), *m*_2_ denotes the mass of the liquid in the outlet pipeline. It should be noted that the pipeline is often long, the liquid in the piezoelectric pump system is mainly distributed in the inlet and outlet pipelines. Equation (4) suggests that the dynamic load of the liquid is proportional to the square of the frequency *f*. Moreover, the dynamic load is also affected by liquid mass and liquid vibration amplitude. Accordingly, at the high driving frequency of piezoelectric pump, the dynamic load of liquid in the outlet pipeline will surge, thereby seriously reducing the output performance of the piezoelectric pump.

**Figure 4:**
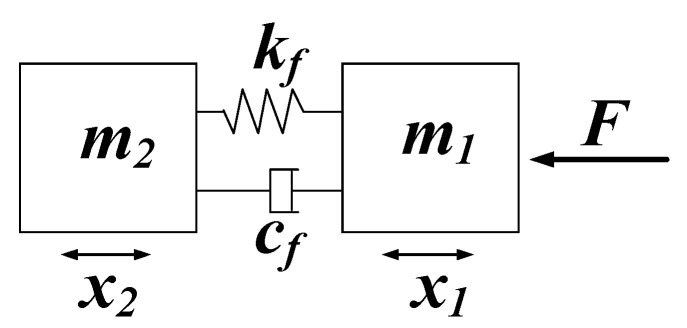
Simplified dynamic model of the piezoelectric pump with elastic inlet and outlet.

When an elastic cavity is added outside the outlet check valve, the elastic cavity cuts off the rigid connection between the liquid in the pump chamber and the liquid in the outlet pipeline. Accordingly, when the piezoelectric pump with elastic inlet and outlet discharges liquid, the vibration model of the pump system can be simplified to the model (Figure 4). The driving force of the piezoelectric actuator is expressed as *F*, the liquid mass in the pump chamber is denoted as *m*_1_, and the liquid mass in the outlet pipeline is *m*_2_. In the model shown in Figure 4, the elastic cavity is expressed by elastic element *k_f_* and damping element c_f_. *x*_1_ refers to the vibration displacement of the liquid in the pump chamber, and *x*_2_ denoted the vibration displacement of the liquid in the outlet pipeline. As revealed from the force analysis of the liquid in the outlet pipeline, it is subject to elastic force of the elastic element *k_f_* and damping force of the damping element c_f_. Following D’Alembert’s principle, the motion differential equation of the liquid in the outlet pipeline is:

(5) $$m_{2}\overset{••}{x_{2}}+c_{f}(\overset{•}{x_{2}}-\overset{•}{x_{1}})+k_{f}(x_{2}-x_{1})=0$$

Next, Equation (5) can be modified to:

(6) $$m_{2}\overset{••}{x_{2}}+c_{f}\overset{•}{x_{2}}+k_{f}x_{2}=k_{f}x_{1}+c_{f}\overset{•}{x_{1}}$$

The elastic cavity slightly impacts the vibration of the liquid in the pump chamber, so *X*_1_ = *x*_1_. Equation (7) can be obtained by substituting Equation (1) into Equation (6).

(7) $$m_{2}\overset{••}{x_{2}}+c_{f}\overset{•}{x_{2}}+k_{f}x_{2}=k_{f}\Phi_{1}\sin(2\pi ft)+c_{f}\Phi_{1}(2\pi f)\cos(2\pi ft)$$

According to the theory of vibration [31], the vibration expression of the liquid in the outlet pipeline can be obtained from Equation (7):

(8) $$x_{2}=\Phi_{1}H\sin(2\pi ft-\varphi )$$

where $\zeta =\frac{c_{f}}{2m_{2}}$, $\lambda =\frac{f}{f_{n}}$, $f_{n}=\sqrt{\frac{k_{f}}{m_{2}}}$, $H=\sqrt{\frac{1+{\left(2\zeta \lambda \right)}^{2}}{{\left(1-\lambda^{2}\right)}^{2}+{\left(2\zeta \lambda \right)}^{2}}}$, $\phi =tg^{-1}\frac{2\zeta \lambda }{1-\lambda^{2}}-tg^{-1}(2\zeta \lambda )$.

ζ is the damping ratio and *λ* is the frequency ratio. *f_n_* is the natural vibration frequency of piezoelectric pump system. *H* is the amplitude amplification factor. *φ* is the lagging phase angle.According to the theory of mechanical vibration, *H* < 1 when *λ* satisfies *λ* > $\sqrt{2}$. Equation (8) shows that the vibration amplitude is reduced and the vibration has a delayed phase angle. Therefore, the vibration of the liquid in the outlet pipeline is smoothed.

The dynamic load of the liquid in the outlet pipeline can be obtained from Equation (8):

(9) $$\bar{F_{2}}=m_{2}\overset{••}{x_{2}}=m_{2}\Phi_{1}H{\left(2\pi f\right)}^{2}\sin(2\pi ft-\varphi )$$

By comparing Equations (4) and (9), the dynamic load reduction factor can be obtained:

(10) $$\frac{\bar{F_{2}}}{F_{2}}=\frac{A_{2}}{A_{1}}H$$

We can easily find that A2/A1 << 1. H is the amplitude amplification factor. According to the theory of vibration mechanics and the design concept of the elastic cavity, we can know that H < 1 in our paper. So the dynamic load reduction factor is much less than 1. This result shows that the dynamic load is effectively reduced.

The frequency ratio *λ* satisfies λ>>1 when the driving frequency *f* of piezoelectric actuator is high. In this regard, the amplitude amplification factor satisfies $H\approx \frac{1}{\lambda^{2}}$. Equation (9) can be modified to:

(11) $$\bar{F_{2}}\approx m_{2}\Phi_{1}\frac{1}{\lambda^{2}}{\left(2\pi f\right)}^{2}\sin(2\pi ft-\varphi )=k_{f}\Phi_{1}\sin(2\pi ft-\varphi )$$

Equation (11) indicates that the dynamic load $\bar{F_{2}}$ is nearly unaffected by the mass *m*_2_ of the liquid in the outlet pipeline and the driving frequency *f*. When the driving frequency *f* is high and the stiffness *k_f_* of the elastic element is small, there is $\bar{F_{2}}<<F_{2}$. Thus, the elastic cavity effectively reduces the dynamic load of the liquid in the outlet pipeline.

## 4. Effect of the Elastic Cavity Height on the Performance of Piezoelectric Pump

Since the height is an important structural parameter of the elastic cavity, choosing the right height will help to give full play to the function of the elastic cavity. In this paper, the effect of the elastic cavity height on the output performance of the piezoelectric pump is studied from both fluid simulation and theoretical analysis.

### 4.1. Fluid Simulation

**Figure 5:**
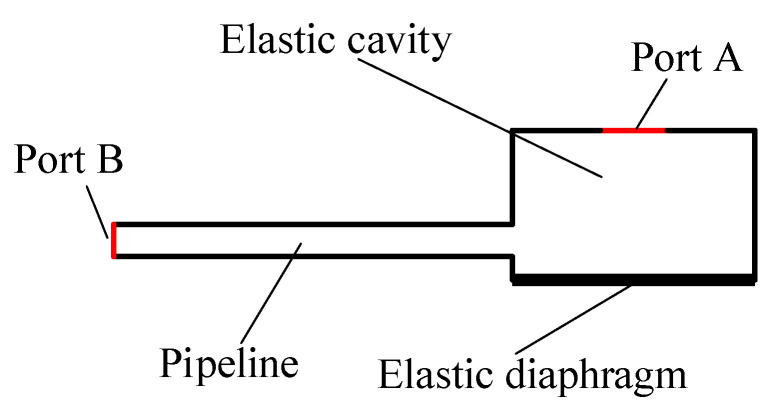
The two-dimensional simulation models.

A two-dimensional fluid-structure coupling simulation of the fluid motion in the elastic cavity was performed using COMSOL software. The two-dimensional simulation model is shown in Figure 5, where port A is the connecting port between the elastic cavity and the check valve and port B is the inlet and outlet of the pipeline. The fluid medium in the simulation is water. In the 2D model, the parameters of the elastic diaphragm are 0.2 mm in thickness and 5 mm in diameter. Its material was defined as silica gel. The two ends of the elastic diaphragm were fixedly connected with the inner wall of the elastic cavity.

To simplify the simulation, the pressure of port A and port B are assumed to be constant.

**Figure 6:**
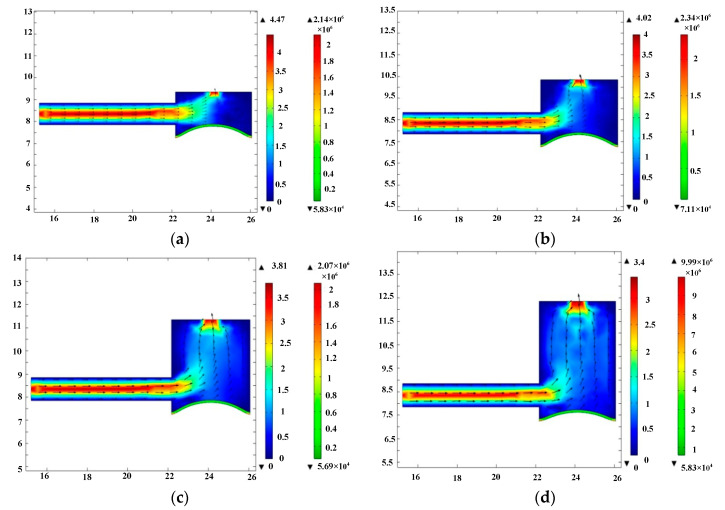
The simulation results of elastic inlet cavity. (**a**) Elastic inlet cavity with height of 2 mm, (**b**) elastic inlet cavity with height of 3 mm, (**c**) elastic inlet cavity with height of 4 mm, and (**d**) elastic inlet cavity with height of 5 mm. The metric for color bar: left color bar-m/s, right color bar-pa.

**Figure 7:**
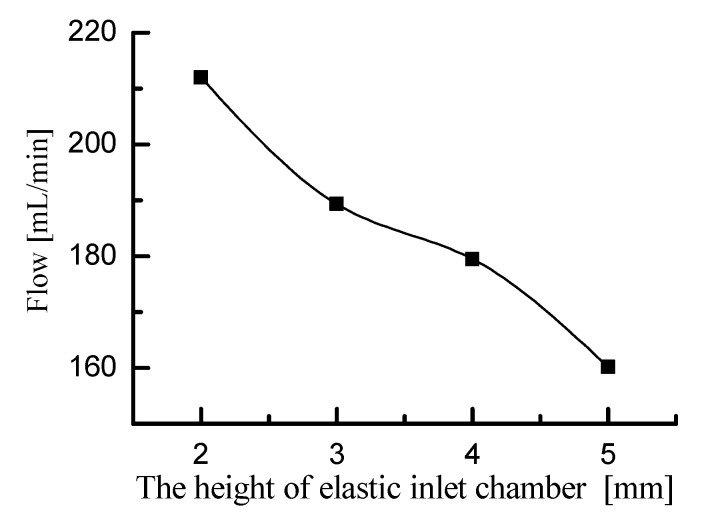
The flow rate at port A corresponding to different heights of elastic inlet cavity.

First, the fluid motion within the elastic inlet cavity was simulated. Port A in the 2D model was defined as the fluid outlet of the elastic inlet cavity. And port B in the 2D model was defined as the fluid inlet of the inlet pipeline. Since the pump chamber draws fluid from the elastic inlet cavity, the pressure at port A should be negative. In the simulation, the pressure at port A was set to −15 kPa. This simulation is only a qualitative simulation of fluid motion trend, so the pressure at port A can also be set to another negative value. Since the inlet pipeline directly absorbs water from the open water tank, the pressure at port B should be 0. The material of the elastic diaphragm was defined as silicone rubber. The fluid medium was defined as pure, incompressible water. Fluid-structure coupling simulations were performed for elastic inlet cavity with heights of 2 mm, 3 mm, 4 mm, and 5 mm, respectively. The simulation results are shown in Figure 6. The simulation results show the flow velocity and streamline of the liquid, as well as the deformation and stress of the elastic diaphragm. Based on the velocity cloud in Figure 6, the flow rate at port A was calculated and is shown in Figure 7. Figure 7 shows that the flow rate at port A decreases as the height of the elastic inlet cavity increases. Considering that the pressure boundary conditions at ports A and B are constant, Figure 7 indicates that the head loss in the elastic inlet cavity increases with the increase of cavity height. Therefore, increasing the height of the elastic inlet cavity is not conducive to the pump to absorb water.

**Figure 8:**
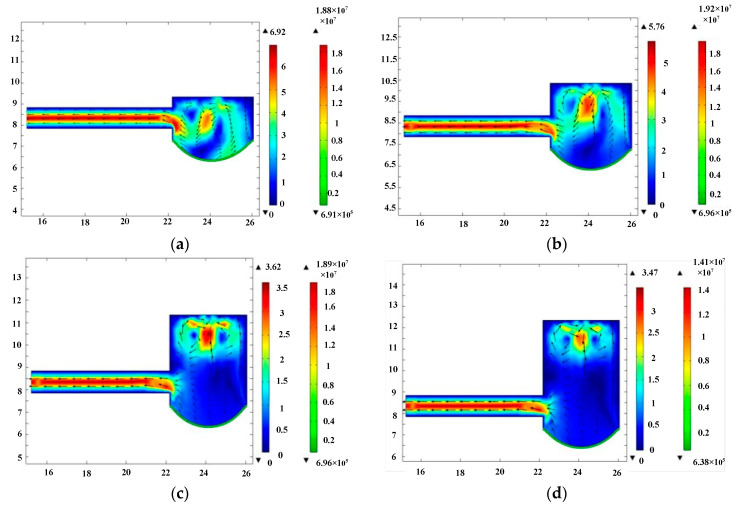
The simulation results of elastic outlet cavity. (**a**) Elastic outlet cavity with height of 2 mm, (**b**) elastic outlet cavity with height of 3 mm, (**c**) elastic outlet cavity with height of 4 mm, and (**d**) elastic outlet cavity with height of 5 mm. The metric for color bar: left color bar-m/s, right color bar-pa.

**Figure 9:**
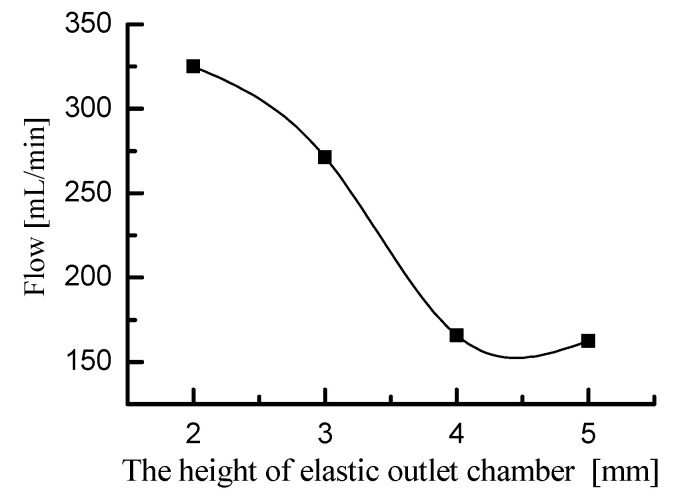
The flow rate at port B corresponding to different heights of elastic outlet cavity.

The fluid motion within the elastic outlet cavity was then simulated. Port A and port B were defined as the fluid inlet and fluid outlet of the 2D model, respectively. Taking into account the inflow and outflow characteristics of the water in the elastic outlet cavity, the pressure at port A and port B should be set to positive pressure and zero pressure, respectively. We set the pressure at port A and port B to 15 kPa and 0, respectively. The settings of the elastic diaphragm and the fluid medium were the same as those in the simulation of the elastic inlet cavity. Fluid-structure coupling simulations were performed for the elastic outlet cavity with heights of 2 mm, 3 mm, 4 mm, and 5 mm, respectively. The simulation results are shown in Figure 8. The flow rate at port B was calculated as shown in Figure 9. Figure 9 shows that the flow rate at port B decreases with the height of elastic outlet cavity. The test result indicates that the head loss in the elastic outlet cavity increases with the cavity height. As a result, the output performance of the piezoelectric pump decreases as the height of the elastic outlet chamber increases. It should be noted that there is no special consideration for setting the pressure at port A to 15 kPa, and its value can also be set to another positive value.

It should be noted that the simulation is a static simulation. The purpose of the simulation is to qualitatively to study the influence trend of the cavity height on the head loss. This qualitative influence trend can be obtained from both dynamic simulation and static simulation. In order to simplify the simulation work, we choose the static simulation in the paper. The pressure at port A was set to a constant value, so this simulation cannot accurately simulate actual working flow rate of the pump. However, this simulation does not hinder the qualitative study of the influence trend of cavity height on head loss.

### 4.2. Theoretical Analysis

**Figure 10:**
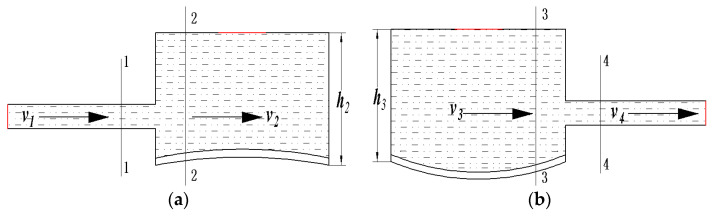
The schematic diagrams of fluid flow in the elastic inlet and outlet cavities. (**a**) Fluid flow in the elastic inlet cavity; (**b**) fluid flow in the elastic outlet cavity.

The fluid flow diagrams of the elastic inlet and elastic outlet cavities are shown in Figure 10. Figure 10a is a schematic representation of the fluid flow from the inlet pipeline to the elastic inlet cavity. Obviously, the fluid flow from the inlet pipeline to the elastic inlet cavity can be viewed as a pipe flow with a sudden increase in cross-section. In Figure 10a, *A*_1_ is the area of cross section 1-1, *A*_2_ is the area of cross section 2-2, *v*_1_ is the flow velocity of the liquid in the inlet pipeline, and *v*_2_ is the horizontal velocity component of the liquid in the elastic inlet cavity. Figure 10b is a schematic representation of the fluid flow from the elastic outlet cavity to the outlet pipeline. Clearly, the fluid flow from the elastic outlet cavity to the outlet pipeline can be considered as a pipe flow with an abrupt decrease in cross-section. In Figure 10b, *A*_3_ is the area of cross section 3-3, *A*_4_ is the area of cross section 4-4, *v*_3_ is the horizontal velocity component of the liquid in the elastic outlet cavity, and *v*_4_ is the flow velocity in the outlet pipeline. According to the local energy loss theory of pipe flow, when the liquid flows from the inlet pipeline to the elastic inlet cavity, the local head loss is:

(12) $$h_{\zeta 2}={\left(1-\frac{A_{1}}{A_{2}}\right)}^{2}\frac{v_{1}^{2}}{2g}$$

As liquid flows from the elastic outlet cavity to the outlet pipeline, the local head loss is:

(13) $$h_{\zeta 3}=0.5(1-\frac{A_{4}}{A_{3}})\frac{v_{4}^{2}}{2g}$$

The areas of cross-sections 2-2 and 3-3 are calculated by Equations (14) and (15), respectively. In Equations (14) and (15), K is a constant value.

(14) $$A_{2}=Kh_{2}$$

(15) $$A_{3}=Kh_{3}$$

Equation (16) can be obtained by Equations (12) and (14). Equation (17) can be obtained by Equations (13) and (15).

(16) $$h_{\zeta 2}={\left(1-\frac{A_{1}}{Kh_{2}}\right)}^{2}\frac{v_{1}^{2}}{2g}$$

(17) $$h_{\zeta 3}=0.5(1-\frac{A_{4}}{Kh_{3}})\frac{v_{4}^{2}}{2g}$$

According to Equations (16) and (17), $h_{\zeta 2}$ decreases as *h*_2_ increases and $h_{\zeta 3}$ decreases as *h*_3_ increases. Therefore, the higher the height of the elastic cavity, the greater the local head loss of the fluid flow. The theoretical analysis is in general agreement with the above simulation analysis.

## 5. Prototype Fabrication and Experimental Device

### 5.1. Experimental Prototypes

**Figure 11:**
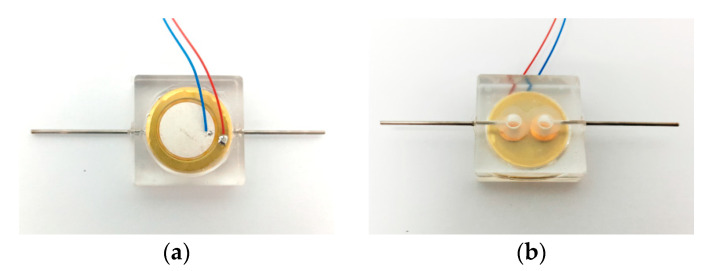
Photo of the prototype with elastic inlet and outlet, (**a**) top view; (**b**) bottom view.

**Figure 12:**
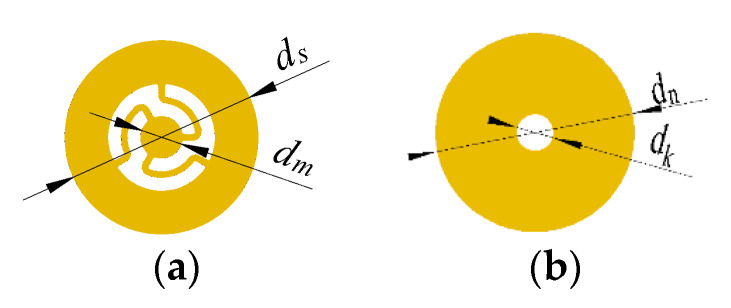
Structure of the wheeled check valve; (**a**) wheeled valve piece; (**b**) valve plate.

**Figure 13:**
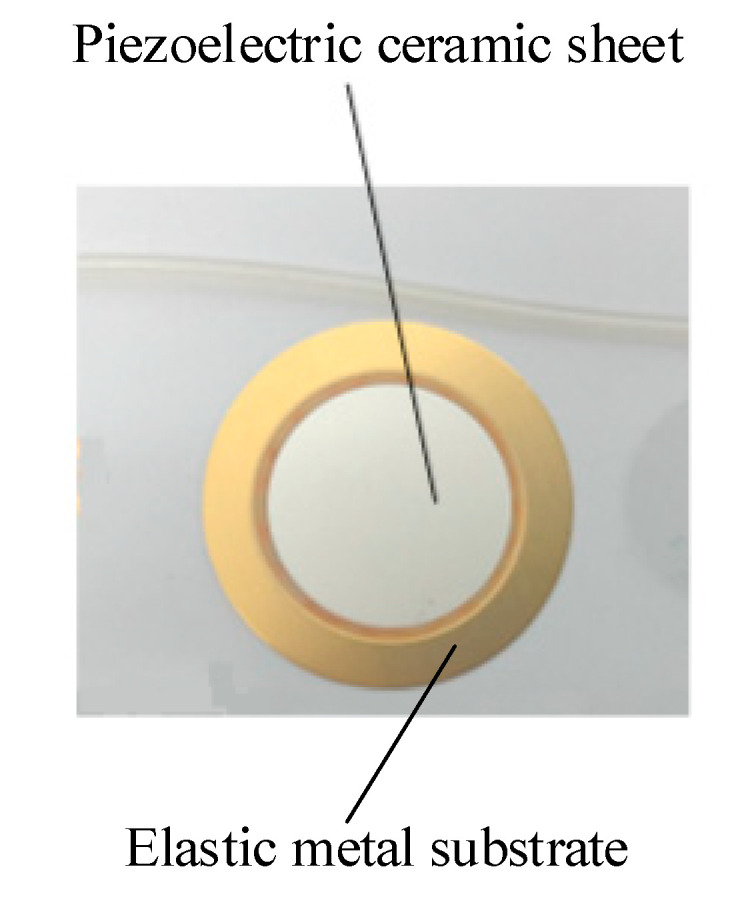
Photo of the piezoelectric vibrator.

To test the effect of the elastic cavity height on the output performance of the piezoelectric pump, four prototypes with elastic cavity heights of 2 mm, 3 mm, 4 mm and 5 mm were fabricated. Except for the height of the elastic cavity, the four prototypes had the same structural parameters. In addition, a prototype without elastic inlet and outlet was fabricated and compared to the prototype with elastic inlet and outlet. The photo of the prototype with elastic inlet and outlet is shown in Figure 11, and the main structural parameters are shown in Table 1. The pump structure is rectangular. The material of the pump body is polymethyl methacrylate (PMMA), which is highly transparent. The inlet and outlet of the pump chamber employ wheel check valves. The construction of the wheel check valve is shown in Figure 12. The wheel check valve is composed of a wheel valve piece and a valve plate. The valve piece and valve plate were made of beryllium bronze. We used laser cutting technology to make these valves. The elastic diaphragm is made of silicone. In Figure 12, *d_s_* is the outside diameter of the wheel check valve plate, *d_m_* is the outside diameter of the moving disk on the wheel check valve plate, *d_n_* is the outside diameter of the valve plate, and *d_k_* is the diameter of the center hole of the valve plate. The piezoelectric vibrator is round. The piezoelectric vibrator was made of elastic metal substrate and piezoelectric ceramic sheet, as shown in Figure 13. The elastic metal substrate and piezoelectric ceramic sheet were glued together by polyethylene terephthalate (PET) glue.

### 5.2. Experimental Device

**Figure 14:**
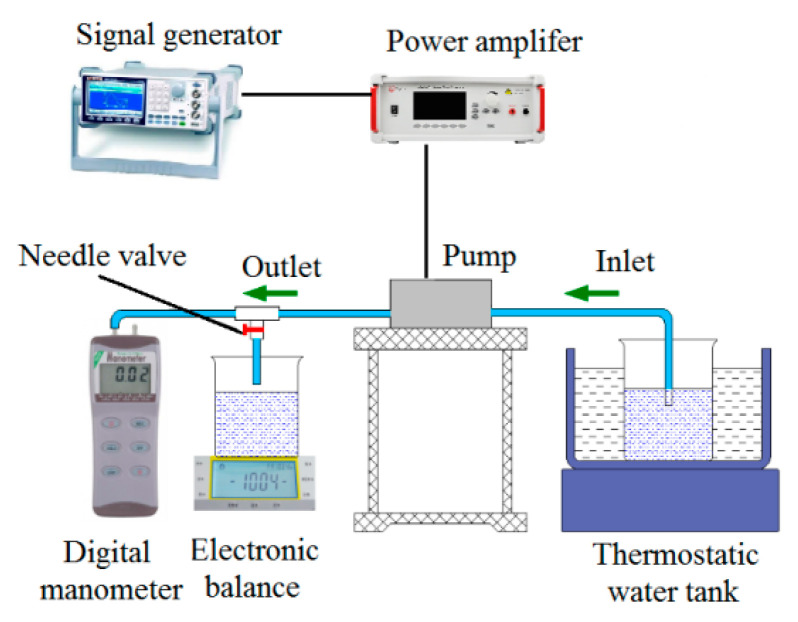
Schematic illustration of the experimental device.

The experimental setup for testing the performance of the prototypes is shown in Figure 14. The signal generator (Rigol, DG 1022) generates a sinusoidal drive signal with a phase shift of 0, which is amplified by a power amplifier (Apex PA94) to actuate the piezoelectric sheet. The voltage amplitude of sinusoidal drive signal is 150 V. The water, which was the pumping medium, was heated to 60 °C and kept at a constant temperature by means of a thermostatic water bath. The purpose of heating and keeping water warm is to eliminate air bubbles in the water and improve the elastic modulus of water. The inlet and outlet pipelines were placed horizontally on the workbench so that the pump has zero backpressure. The flow rate of the piezoelectric pump was measured using the weighing method and the output pressure was measured using a digital manometer. In order to reduce measurement errors in the experimental data, four measurements were taken for each prototype and the mean value was calculated.

## 6. Results and Analysis

### 6.1. Performance Improvement of Piezoelectric Pump by Elastic Inlet and Outlet

**Figure 15:**
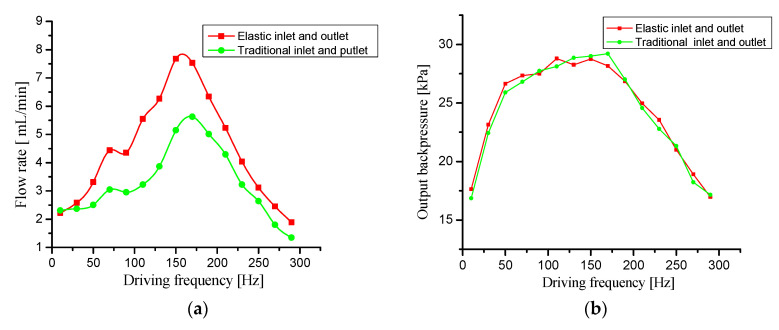

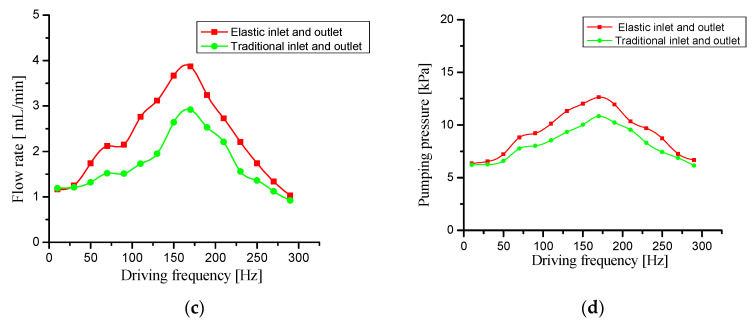
Performance comparison test curves of the prototypes with and without elastic inlet and outlet. (**a**) Flow rate—driving frequency curves with needle valve fully open, (**b**) output backpressure—driving frequency curves with needle valve fully closed, (**c**) flow rate—driving frequency curves with 50% needle valve opening, and (**d**) output backpressure—driving frequency curves with 50% needle valve opening.

First, the output backpressure and flow rate of the prototype without elastic inlet and outlet were tested at different drive frequencies. Then, the output backpressure and flow rate of the prototype with elastic inlet and outlet (2 mm high elastic cavity) at different drive frequencies were tested. The test results are shown in Figure 15. Figure 15a shows flow comparison curves of the two tested prototypes when the needle valve is fully open. Figure 15b shows backpressure comparison curves when the needle valve is fully closed. Figure 15c,d respectively shows flow comparison curves and backpressure comparison curves when the needle valve opening is 50%.

Comparing the flow rate curves of the two prototypes in Figure 15a, we can see that the flow rate of the prototype with elastic inlet and outlet is significantly higher than that of the prototype without elastic inlet and outlet. The prototype with elastic inlet and outlet has a 36% higher maximum flow rate than the prototype without the elastic inlet and outlet. When the driving frequency *f* is low (*f* < 30 Hz), there is no significant difference in the output flow between the two prototypes.The reason for this phenomenon is that the elastic cavity does not work due to the low frequency ratio *λ*. When the driving frequency *f* increases gradually (30 < *f* < 170 Hz), the flow rate curve of the prototype with elastic inlet and outlet rises rapidly, which is significantly higher than that of the prototype without elastic inlet and outlet. This phenomenon indicates that the elastic cavity works and reduces the dynamic load. When the driving frequency *f* reaches about 180 Hz, the flow rate of the two prototypes reaches the peak value, which shows that the resonant frequency of the prototypes is about 180 Hz. When the driving frequency continues to increase (*f* *>* 180 Hz), the flow rate of the prototype with inlet and outlet is still higher than that without inlet and outlet, but the flow difference is gradually reduced. Therefore, when selecting the driving frequency of the piezoelectric pump with elastic inlet and outlet, it should be less than the resonance frequency. Comparing the flow rate curves in Figure 15c, we can find that the change trend of the flow rate curves is similar to that in 15a. This result shows that the elastic cavity can also effectively improve the flow rate when the pump system load is not 0.

Comparing the output backpressure curves of the two prototypes in Figure 15b, it is clear that there is no significant difference between them. The output backpressure in Figure 15b was tested at zero flow rate and was the maximum output backpressure of the two prototypes. In the output backpressure test at zero flow rate, the liquid in the pipelines is not flowing, so the dynamic load of the liquid in the pipelines is negligible. At this point, the elastic inlet and outlet do not function, so the maximum output backpressure is nearly identical for the two test prototypes. Comparing the output backpressure curves in Figure 15d, we can see that the output backpressure of the prototype with elastic inlet and outlet is significantly higher than that of the prototype without elastic inlet and outlet when the needle valve opening is 50%. This result shows that the elastic cavity effectively reduces the pressure loss caused by dynamic load and improves the output backpressure.

In summary, the elastic inlet and outlet effectively improve the flow rate and output backpressure of the piezoelectric pump without increasing the maximum output backpressure.

### 6.2. Effect of the Elastic Cavity Height on the Performance of Piezoelectric Pump

**Figure 16:**
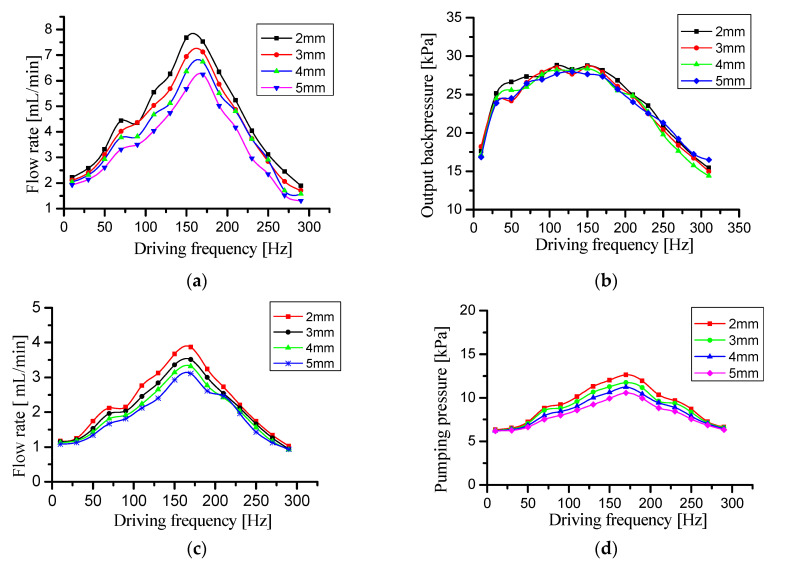
Performance comparison test curves of the four prototypes with elastic cavity heights of 2 mm, 3 mm, 4 mm and 5 mm. (**a**) Flow rate—driving frequency curves with needle valve fully open, (**b**) output backpressure—driving frequency curves with needle valve fully closed, (**c**) flow rate—driving frequency curves with 50% needle valve opening, and (**d**) output backpressure—driving frequency curves with 50% needle valve opening.

Four prototypes with elastic cavity heights of 2 mm, 3 mm, 4 mm, and 5 mm were tested for flow rate and output backpressure, respectively. The test results are shown in Figure 16.

Comparing the flow rate curves of the four prototypes in Figure 16a,c respectively, it can be seen that the higher the height of the elastic cavity, the lower the flow rate of the prototypes. The test result shows that the height of the elastic cavity has a detrimental effect on the flow rate. According to the analysis in 4.1 and 4.2, the higher the height of the elastic cavity, the greater the local head loss in the elastic cavity. Therefore, the higher the height of the elastic cavity, the lower the flow rate of the prototype. Comparing the flow rate curves.

Comparing the output backpressure curves of the four prototypes in Figure 16b, it can be seen that there is no significant difference between the four prototypes. The output backpressure in Figure 16b is the maximum output backpressure of the four prototypes. The test result shows that the height of the elastic cavity has not a detrimental effect on the maximum output backpressure. In the maximum backpressure test at zero flow rate, there is no local head loss in the elastic cavity because the liquid in the pipelines does not flow. So the maximum output backpressure curves of the four prototypes are almost the same. However, comparing the output backpressure curves in Figure 16d, there are obvious differences between the four prototypes. Figure 16d shows that the output back pressure decreases with the height of the elastic cavity. The test result indicates that the height of the elastic cavity also has a detrimental effect on the output backpressure. The reason for this phenomenon is that the local head loss in the elastic cavity increases with the height of the elastic cavity.

In summary, increasing the height of the elastic cavity has a detrimental effect on the flow rate and output backpressure, but has no effect on the maximum back pressure.

## 7. Conclusions

In this study, elastic inlet and outlet are proposed to reduce the dynamic load of liquid in the inlet and outlet pipelines. After theoretical analysis, simulation analysis and prototype testing, the following conclusions can be drawn:

The elastic inlet and outlet could effectively improve the flow rate and output backpressure of piezoelectric pumps. When the piezoelectric pump system had no load, the maximum flow rate was increased by 36%. However, the elastic inlet and outlet could not increase the maximum output backpressure.

The local head loss in the elastic cavity increased with the increase of the height of the elastic cavity. So increasing the height of the elastic cavity had a detrimental effect on the flow rate and output backpressure. However, the height of the elastic cavity had no effect on the maximum output backpressure.

The height of the elastic cavity should be as low as possible in view of the local head loss in the elastic cavity. However, if the height of the elastic cavity is too low, the function of the elastic inlet and outlet will be weakened. The disadvantage of this study is that the optimal height of the elastic cavity was not investigated. In a follow-up study, we will optimize the structure of the elastic cavity to reduce the local head loss or find the best height calculation method.

## Figures and Tables

**Table 1:** The main parameters of prototypes.

Structural Parameters	Values
Diameter and thickness of piezoelectric vibrator	Φ20 × 0.4 (mm)
Diameter and thickness of the metal substrate	Φ20 × 0.2 mm
Diameter and thickness of the piezoelectric ceramic sheet	Φ16 × 0.2 mm
Outer diameter of wheeled valve piece *d_s_*	5 mm
Outer diameter of the moving disc on the wheeled valve piece *d_m_*	1.4 mm
Thickness of wheeled valve piece	0.03 mm
Outer diameter of valve plate *d_n_*	5 mm
Diameter of center hole of valve plate *d_k_*	1.2 mm
Thickness of valve plate	0.05 mm
Height of the pump chamber	0.15 mm
Diameter of inlet and outlet pipeline	1.5 mm
Thicknesses of elastic diaphragm	0.2 mm
Height of elastic cavity	2, 3, 4, 5 mm
Diameter of elastic cavity	3 mm
